# Inappropriate statistical method in a parallel-group randomized controlled trial results in unsubstantiated conclusions

**DOI:** 10.1186/s12937-016-0163-z

**Published:** 2016-06-06

**Authors:** Rositsa B. Dimova, David B. Allison

**Affiliations:** 1 Institute of Nutrition, ltu, São Paulo Brazil; 2 Department of Medicine, Division of Medical Nutrition, Ribeirão Preto Medical School, University of São Paulo, Avenida Bandeirantes, 3900 Bairro Monte Alegre, CEP: 14049-900, Ribeirão Preto, São Paulo Brazil

**Keywords:** Statistical analyses, Flaxseed, Research integrity, Research reporting, Nutrition, Obesity

## Abstract

The conclusions of Cassani et al. in the January 2015 issue of *Nutrition Journal* (doi:10.1186/1475-2891-14-5) cannot be substantiated by the analysis reported nor by the data themselves. The authors ascribed the observed decrease in inflammatory markers to the components of flaxseed and based their conclusions on within-group comparisons made between the final and the baseline measurements separately in each arm of the randomized controlled trial. However, this is an improper approach and the conclusions of the paper are invalid. A correct analysis of the data shows no such effects.

## Letter to the editor

Randomized controlled trials (RCTs) are vital to estimating and testing the causal effects of treatments but are only as good as their design, execution, analysis, and reporting [1]. Rigorous assessment of the effects of foods such as flaxseed on health outcomes is informative and useful [2]. Hence, Cassani et al.’s [3] description of an RCT of the effects of flaxseed consumption on body weight and inflammation markers is of interest. Regrettably, however, the authors draw conclusions that cannot be supported by the statistical analyses used. Moreover, discrepancies between the published paper and clinical trial registration raise questions.

### Discrepancies between the published paper and clinical trial registration

The study registration on ClinicalTrials.gov shows that the study was completed in 2007 and yet registered in 2014. Moreover, it was designed as a four-arm study, yet the article reports results from only two groups and offers neither explanation for nor mention of this discrepancy. This seems inconsistent with the transparency that clinical trials registration is designed to promote [4].

## Findings

### The erroneous nature of the analyses

Cassani et al concluded that “flaxseed added to a weight loss diet could be an important nutritional strategy to reduce inflammation markers such as CRP and TNF- α.” The statistical analyses offered in the article, however, were not legitimate tests of the hypotheses in question. The statistical analyses utilized entailed comparisons of the outcomes measured at the end of the study with their pre-intervention levels in each arm separately. The abstract states, “A decrease in inflammatory markers (CRP and TNF-α) was observed after flaxseed intake (mean decrease of 25 % and 46 % for G_flaxLC_ respectively). All groups also showed improvement in levels of total cholesterol, LDL-c, uric acid and adiponectin.” The main reason for conducting an RCT is to provide comparison of an outcome of interest between two or more experimental groups or conditions to which subjects have been randomly assigned, such that *expected* distributions of subject characteristics before administering the intervention (s) are identical across randomized groups or conditions [5]. Drawing inferences based on the detected (or not) differences within a group is improper and can lead to inaccurate and misleading conclusions [6]. As Bland and Altman [6] show, this approach can produce type 1 error rates of up to 0.50 for two-group comparisons when the 0.05 alpha level is used, in other words, a possible inflation of the false-positive rate by up to an order of magnitude. Instead, the analysis should include direct comparison of the outcome measured at the end of the study *between* the groups, usually adjusted for the pre-intervention levels.

By omitting direct between-group comparison of the outcome variables and by relying on the within-group results, Cassani et al.’s article misinforms. Specifically, the article states that a significant decrease in the inflammatory markers CRP and TNF- α was observed only in the flaxseed group. The authors thus concluded that adding flaxseed to a weight loss diet could reduce the inflammation markers CRP and TNF- α. However, no comparison between the two groups was provided. Thus, the authors did not answer the question of whether there was a significant difference in the outcomes between the two interventions.

### What does a proper analysis of between-group differences show?

A proper analysis of these data could take several forms, such as a between-groups *t*-test (or nonparametric test) on the endpoint data or on the change-scores, or a between-groups ANCOVA on the endpoint with baseline values as a covariate, as described by Allison et al. [7]. All of these involve a test *between* the randomized groups.

Cassani et al. should be commended for making their raw data publically available and for noting this in their article. We downloaded their data, which were then declared deidentified non-human subjects data by the University of Alabama at Birmingham’s Institutional Review Board. We calculated the mean (SD) baseline and final CRP levels to be respectively 2.77 (2.45) and 1.99 (2.07) in the G_riceLC_ group, and 2.04 (1.48) and 1.51 (0.92) in the G_flaxLC_ group. An ANCOVA of the final log-transformed CRP levels adjusted for baseline log-transformed CRP levels suggests no significant difference between the two groups (*p*=0.94)^1^. Data for TNF-α were available for only 9 (G_riceLC_) and 11 (G_flaxLC_) subjects, and contained 2 apparent outliers of 3446 and 1140, which we assumed to correspond to 34.46 and 11.40. Accordingly, we calculated the mean (SD) baseline and final TNF-α levels to be 25.67 (16.00) and 22.31 (17.17), respectively, in the G_riceLC_ group, and 15.46 (16.39) and 7.67 (10.05), respectively, in the G_flaxLC_ group. An ANCOVA of the final TNF-α levels adjusted for baseline TNF-α levels suggests no significant difference between the two groups (*p* = 0.09)^2^. Removal of the outliers also produced nonsignificant results. Alternatively, a rank-sum test of the change-score between the two groups, for both CRP and TNF-α, produces nonsignificant results (*p* = 0.81 and *p* = 0.82, respectively). Additionally, it should be pointed out that the difference in the baseline levels between the two groups, and correctness of the data should be assessed, especially given the small sample size.

## Conclusions

Those interested in evidence-based recommendations need evidence from sound scientific studies, including soundness of design, execution, analysis, and reporting [8] without “spin” [9]. According to the Committee on Publication Ethics [10], “Journal editors should consider retracting a publication if…they have clear evidence that the findings are unreliable [including]… as a result of …miscalculation or experimental error.” Cassani et al.’s [3] conclusions are not reliable because an invalid statistical testing approach was used, and retraction should be considered.

## Endnotes

^1^Levene’s test for homogeneity of variance of the final log-transformed CRP levels was insignificant, *p* = 0.18.

^2^Levene’s test for homogeneity of variance of the final TNF-α levels was insignificant, *p*=0.29.

## Competing interests

The authors report no financial connection to the content of the paper discussed. David B. Allison and/or his institution have accepted funds from food companies, but not ones who, to his knowledge, market flaxseed.

## Authors’ contributions

DBA conceived the paper. Both authors drafted sections of the manuscript and edited the entire paper. Both authors read and approved the final manuscript.

## Author details

^1^Department of Biostatistics, University of Buffalo, Buffalo, NY 14214, USA. ^2^Office of Energetics and Nutrition Obesity Research Center, University of Alabama at Birmingham, School of Public Health, Ryals Public Health Building, Room 140J, Birmingham, AL 35294, USA.

## Acknowledgements

Supported in part by NIH grants P30DK056336, R25DK099080, and R25HL124208. The content is solely the responsibility of the authors and does not necessarily represent the official views of the National Institutes of Health or any other organization. The authors thank Drs. Peng Li, Brandon George, and Mark B. Cope for constructive input.
