# Supplementary material for: Inappropriate statistical method in a parallel-group randomized controlled trial results in unsubstantiated conclusions
Source: Nutr J. 2016 Jun 6;15:58. doi: 10.1186/s12937-016-0163-z (PMC4893839; doi:10.1186/s12937-016-0163-z)
Supplement: Related texts

# Reply: Response to “Inappropriate statistical method in a parallel-group randomized controlled trial results in unsubstantiated conclusions”

**Authors:** Roberta Soares Lara Cassani, Priscila Giacomo Fassini, Jose Henrique Silvah, Cristiane Maria Mártires Lima, Júlio Sérgio Marchini

**Affiliations:** 3 Institute of Nutrition, ltu, São Paulo Brazil; 4 Department of Medicine, Division of Medical Nutrition, Ribeirão Preto Medical School, University of São Paulo, Avenida Bandeirantes, 3900 Bairro Monte Alegre, CEP: 14049-900, Ribeirão Preto, São Paulo Brazil

## Discrepancies between the published article and clinical trial registration records

Dimova and Allison have raised concerns about discrepancies between our published article and the clinical trial registration records. The first relates to the date of the trial registration. Although this study was completed in 2007, our group only started to register our studies in ClinicalTrials.gov in 2014. However, the trial was registered in the database of brazilian studies (CAAE - Certificado de Apresentação para Apreciação Ética - Certificate of Presentation for Ethical Consideration 0161.0.004.000-07) before the work began.

The other discrepancy raised was the absence of two groups in the published study compared with the four groups in the clinicaltrials.gov record. This was a result of suggestions from reviewers.

The reviewer for another journal, to which this article was submitted before submission to the *Nutrition Journal*, recommended that one of the four groups should be not included in the article. Differences between the subjects in this group and the other three groups were given as the reason for its exclusion. Later, one of the reviewers for the *Nutrition Journal* requested that another group should be removed because of the different intervention used in this group, compared with the two groups in the published article.

## Erroneous findings

After reading the article by Bland and Altman [11] and reviewing the statistical analyses, all authors agree that this study does not allow us to conclude that there was decrease in the blood levels of CRP and TNF-alpha that could be attributed to flaxseed. Initially, the reviewer for the *Nutrition Journal* requested that a between-groups *t*-test or its non-parametric equivalent should be performed on our data. This was done for all the data described in the Tables, which could be noted in the article as the tests used between-groups mean differences. Unfortunately, only now it was noticed that the same was not done in the data shown in the figures (e.g. CRP, TNF-alpha and isoprostane). However, we would like to emphasize that this error was not observed by the *Nutrition Journal*, which accepted our article.

We thank Dimova and Allison for commending us for making our data available to the scientific community. Also, we were not “disappointed that their treatment had not produced a significant difference, as well as had not searched for anything that will have the appearance of supporting it”, as pointed out by Bland and Altman [11].

Finally, by making our data available to the scientific community, we hope that the errors in the statistical analysis will be seen as an honest mistake for which we sincerely apologize.
